# Foliar potassium–calcium nutrition enhances fruit yield, quality and mitigates cracking in guava (*Psidium guajava* L.) under humid subtropical conditions

**DOI:** 10.3389/fpls.2026.1812647

**Published:** 2026-04-07

**Authors:** Vivek Dangwal, Vijay P. Singh, Daya Shankar Mishra, Mandeep Rawat, Krishna, Rajesh Kumar, Prakashbhai Ravat, Ratna Rai, Vikas Yadav, Prabhat Kumar, Gaurav Sharma, Ajay Kumar, Virendra Kumar, Omveer Singh, Rajkumar Jat

**Affiliations:** 1 G.B. Pant University of Agriculture and Technology, Pantnagar, Uttarakhand, India; 2 ICAR-Central Horticultural Experiment Station, Vejalpur, Panchmahals, Gujarat, India; 3 ICAR-Central Institute for Arid Horticulture, Bikaner, Rajasthan, India; 4 ICAR-Indian Agricultural Research Institute, New Delhi, India; 5 Rani Lakshmi Bai Central Agricultural University, Jhansi, Uttar Pradesh, India; 6 School of Agriculture & Development Studies, Uttarakhand Open University, Haldwani, Uttarakhand, India

**Keywords:** foliar nutrient application, fruit cracking, fruit quality, guava, potassium–calcium interaction, yield improvement

## Abstract

**Introduction:**

Guava (*Psidium guajava L*.) productivity and fruit quality are frequently constrained by nutrient imbalances during reproductive development, leading to yield losses and physiological disorders such as fruit cracking. Potassium (K) and calcium (Ca) play critical roles in plant physiological regulation and cell wall stability, yet their coordinated effects on yield, fruit quality, and underlying trait relationships in guava remain insufficiently understood.

**Methods:**

In this study, we evaluated the influence of foliar-applied K and Ca compounds on vegetative growth, leaf nutrient status, fruit yield, quality attributes, and fruit cracking in the high-yielding guava cultivar ‘VNR Bihi’ under subtropical humid conditions. A total of 13 treatments comprising different concentrations of potassium nitrate, potassium sulphate, calcium nitrate, and calcium sulphate, along with an untreated control, were applied at fruit set and one month thereafter.

**Results:**

Foliar nutrient application significantly enhanced leaf growth, chlorophyll content, and post-treatment leaf K and Ca concentrations compared with the control. Yield parameters responded positively to nutrient supplementation, with potassium nitrate at 2.0% producing the highest fruit yield (41.44 kg tree⁻¹; 27.60 t ha⁻¹), accompanied by marked reductions in fruit drop and cracking. Fruit physical and biochemical quality traits, including fruit weight, total soluble solids, sugars, and ascorbic acid, were also improved by K and Ca treatments. Multivariate analyses revealed clear treatment-wise clustering based on fruit retention, drop, and cracking, while partial least squares regression demonstrated a strong predictive relationship between leaf physiological traits and fruit quality (R^2^ = 0.877).

**Discussion:**

Overall, the results indicate that foliar K and Ca application modulates guava yield and fruit quality through coordinated physiological and nutritional responses, providing a trait-based framework for reducing fruit cracking and enhancing productivity.

## Introduction

Guava (*Psidium guajava* L.) is an economically and nutritionally important fruit crop cultivated widely across tropical and subtropical regions. Owing to its high content of vitamin C, dietary fiber, pectin, and bioactive compounds, guava is often referred to as the “apple of the tropics” and plays a significant role in nutritional security and income generation for smallholder farmers (Khan et al., 2013; Nimisha et al., 2013; Takeda et al., 2022; Mishra et al., 2025b; Maan et al., 2023). Guava has emerged as the fourth most important fruit crop in India, occupying an area of 358 thousand hectares and producing 5.35 million metric tonnes, with an average productivity of 15 t ha^-^;¹ (Pramanik et al., 2025). Despite its commercial importance, achieving consistently high yield with superior fruit quality remains a challenge, particularly under subtropical conditions where seasonal variations strongly influence nutrient uptake and fruit development. Among mineral nutrients, potassium (K) and calcium (Ca) are critical regulators of plant growth, metabolism, and fruit development. Potassium functions as a major osmoticum and enzyme activator, contributing to photosynthesis, assimilate translocation, stomatal regulation, and cell expansion, all of which directly influence fruit size, sweetness, and yield (Tiwari, 2005; Kumar et al., 2017; Nessem, 2019; Burhan and AL-Taey, 2018). Calcium, in contrast, plays a structural and signaling role, stabilizing cell walls and membranes, regulating cell division, and modulating hormonal and enzymatic activities associated with fruit firmness and resistance to physiological disorders (Dorria, 2001; Taylor, 2003; Shirzadeh and Kazemi, 2011; Ali et al., 2021). Adequate Ca supply is particularly important for maintaining peel integrity and reducing fruit cracking, a disorder linked to weakened cell walls and imbalanced water relations during fruit growth (Fan et al., 2023; Dong et al., 2025). In fruit crops, the demand for K and Ca increases markedly during flowering and fruit development, often exceeding the capacity of root uptake alone. This limitation is further exacerbated during winter-season cropping under subtropical conditions, when low soil temperatures restrict nutrient mobility and absorption (Fernández et al., 2021). As a result, nutrient deficiencies frequently arise during critical developmental stages, leading to reduced fruit quality, increased fruit drop, and heightened susceptibility to physiological disorders such as fruit cracking (Solhjoo et al., 2017; Wang et al., 2024). Foliar fertilization has therefore emerged as an effective strategy for rapidly correcting nutrient deficiencies by delivering nutrients directly to metabolically active tissues, improving nutrient use efficiency, and minimizing soil-related constraints (Mishra et al., 2003; Amiri et al., 2008; Fageria et al., 2009).

Previous studies have demonstrated beneficial effects of foliar K and Ca applications on yield, fruit quality, and mineral composition in several fruit crops, including apple, pear, citrus, mango, and guava (Solhjoo et al., 2017; EL-Shobaky and Mohamed, 2000; Dhatt et al., 2005; Kumar et al., 2015). Potassium-mediated improvements in sugar accumulation and total soluble solids have been attributed to enhanced phloem loading and assimilate transport, whereas Ca-mediated reductions in fruit cracking are associated with increased pectin cross-linking and reduced activity of cell wall–degrading enzymes (Fan et al., 2023; Dong et al., 2025; Zhu et al., 2023). However, earlier studies have focused primarily on individual traits or single nutrient effects, with limited integration of physiological, nutritional, and multivariate trait relationships. The guava cultivar ‘VNR Bihi’, characterized by large fruit size, high yield potential, and extended shelf life, exhibits higher nutrient demand than traditional cultivars, particularly during the winter cropping season. Soil fertilization alone is often insufficient to meet this demand, leading to compromised fruit quality and increased incidence of fruit cracking and drop. Moreover, the mechanistic links between leaf physiological status, nutrient dynamics, and fruit quality outcomes in guava remain poorly resolved, limiting the development of predictive frameworks for yield and disorder management. Therefore, the present study aimed to (i) evaluate the effects of foliar-applied potassium and calcium compounds on vegetative growth, leaf nutrient status, fruit yield, and quality traits in guava; (ii) assess their role in regulating fruit cracking and fruit drop; and (iii) elucidate the relationships between leaf physiological traits and fruit quality parameters using multivariate approaches, including hierarchical clustering and partial least squares regression. By integrating physiological, nutritional, and statistical analyses, this study seeks to provide a trait-based understanding of K- and Ca-mediated regulation of yield and fruit quality in guava.

## Materials and methods

### Experimental site and plant material

**Figure 1:**
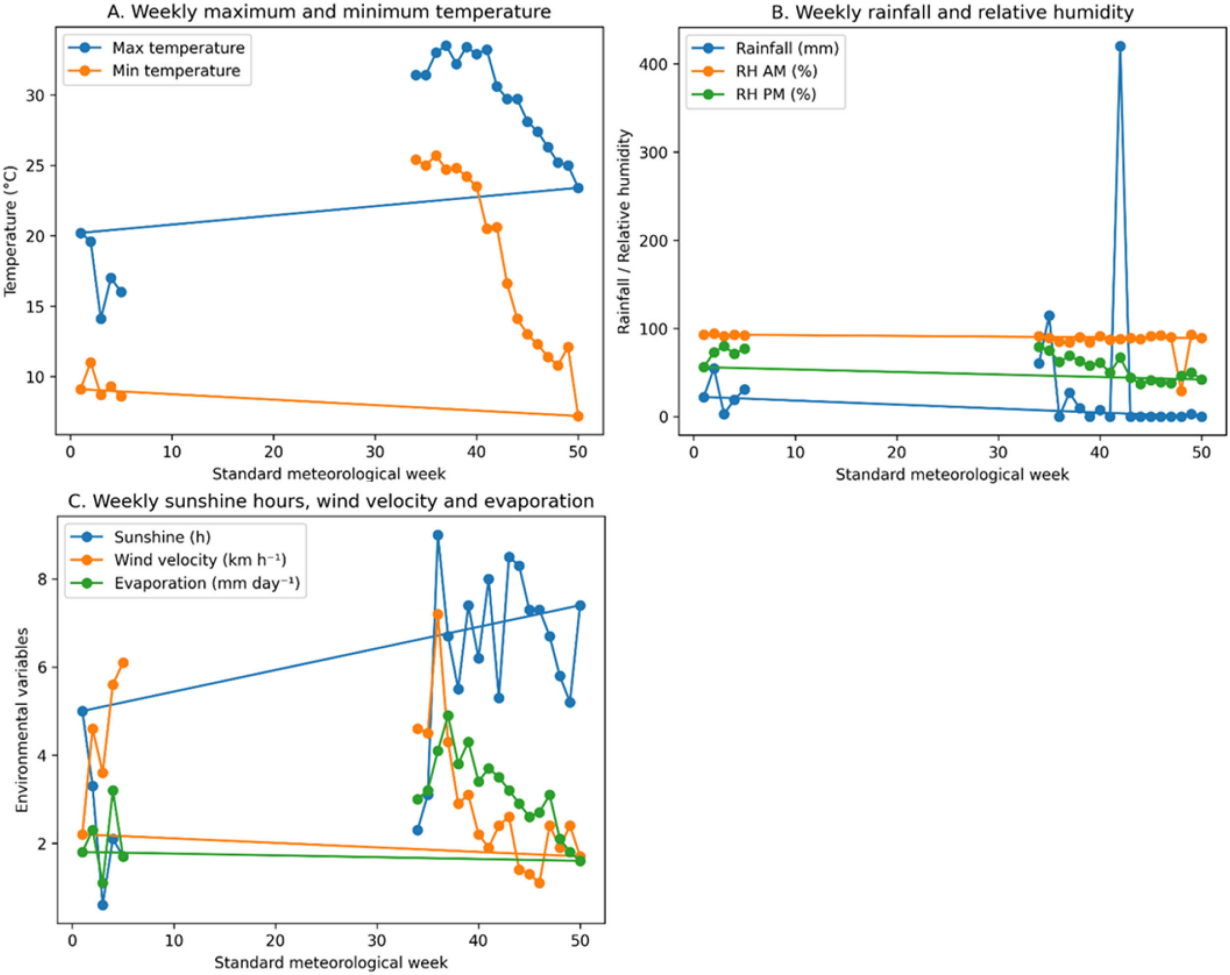
Weekly mean meteorological parameters during the experimental period (2022–23), including rainfall, maximum and minimum temperature, relative humidity, evaporation, and sunshine hours at the experimental site.

The experiment was conducted during the winter cropping season of 2022–23 at the Horticulture Research Centre, Govind Ballabh Pant University of Agriculture and Technology, Pantnagar, Uttarakhand, India (29.02° N, 79.41° E; 243.8 m above mean sea level). The region experiences a subtropical humid climate characterized by hot summers and cold winters. During the experimental period, maximum temperatures ranged from 30 to 43 °C, while minimum temperatures dropped to 1.5 °C in January. The total annual rainfall during the cropping season was 1258 mm, with monsoon precipitation occurring mainly between June and September. Weekly meteorological parameters recorded during the experiment are presented in **Figure 1**.

**Figure 2:**
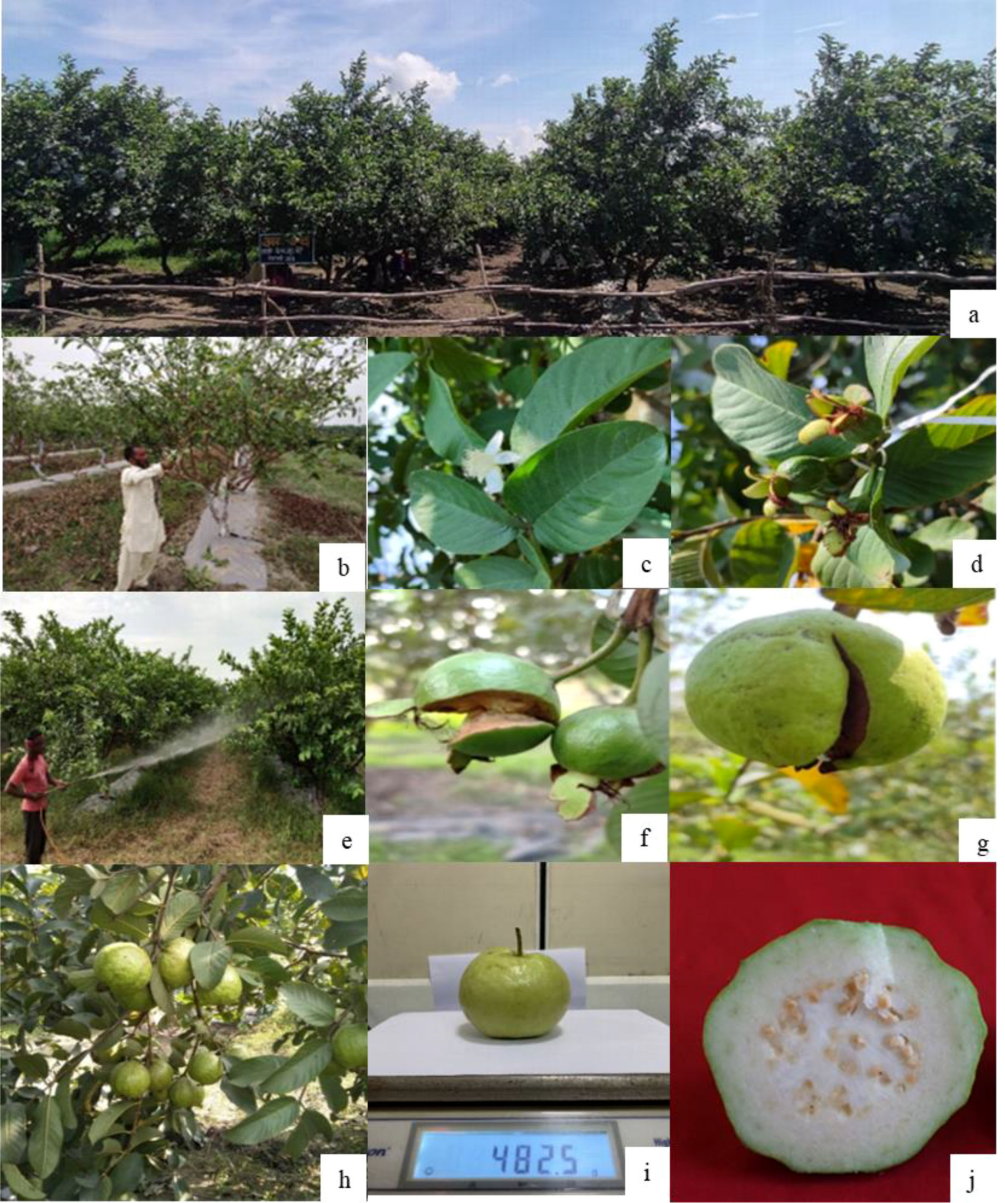
Representative stages of orchard management and fruit development in guava **(a)** experimental guava orchard; **(b)** summer pruning for winter season harvest; **(c)** flowering stage; **(d)** fruit set; **(e)** foliar nutrient application; **(f, g)** fruit cracking in untreated control; **(h)** fruiting stage; **(i)** fruit weight under the best treatment; and **(j)** transverse section of mature fruit.

Seven-year-old, wedge-grafted guava trees of the cultivar ‘VNR Bihi’ were used in the present the study. The guava trees were trained and maintained under an open-center training system to facilitate improved canopy architecture, light interception, and air circulation. The experimental orchard was established at a spacing of 5 m × 3 m under a medium–high-density planting system. A total of thirty-nine uniform and healthy trees were selected and arranged in a randomized block design (RBD) comprising 13 treatments with three replications, each replication consisting of a single tree. The original grafted planting material of cv. ‘VNR Bihi’ was procured from VNR Nursery, Raipur, Chhattisgarh, India, a registered commercial nursery specializing in certified fruit planting material. The cultivar identity was confirmed at the time of establishment based on nursery certification records and standard morphological descriptors of guava by the research team prior to the commencement of the experiment (**Figure 2**).

### Soil characteristics and basal fertilization

Soil samples were collected from the 0–15 cm depth prior to the initiation of the experiment, as this layer represents the most biologically active soil zone and the primary region of nutrient uptake in fertilized orchard systems (Singh et al., 2012). The samples were then analyzed for their physicochemical properties. Soil samples were collected from five randomly selected locations within the orchard and pooled to obtain a representative composite sample. The soil was classified as silt loam with neutral pH (7.1) and moderate fertility status. Available nitrogen, phosphorus, potassium, calcium, and sulphur were determined following standard procedures (Subbiah and Asija, 1956; Olsen, 1954; Jackson, 1973). Soil chemical properties are summarized in **Table 1**. All experimental trees received uniform basal fertilization as per regional recommendations. Nitrogen (450 g tree^-^;¹), phosphorus (390 g tree^-^;¹), and potassium (300 g tree^-^;¹) were applied through urea, diammonium phosphate, and muriate of potash, respectively. The full dose of phosphorus and potassium and half of the nitrogen were applied in mid-June, while the remaining nitrogen was applied in September.

**Table 1:** Soil chemical properties of the experimental orchard site prior to foliar application (0–15 cm depth).

Organic C (%)	pH	EC (mmoh cm^-1^)	N (kg ha^-1^)	P (kg ha^-1^)	K (kg ha^-1^)	Ca (kg ha^-1^)	S (kg ha^-1^)
0.67	7.1	0.19	186.49	20.47	155.8	15.34	16.81

### Treatment details and foliar application

Thirteen treatments were imposed, comprising foliar application of potassium nitrate (KNO_3_) and potassium sulphate (K_5_4;) at 1.0, 2.0, and 3.0%, and calcium nitrate [Ca(NO_3_)_2_] and calcium sulphate (CaSO4;) at 0.5, 1.0, and 1.5%, along with an untreated control receiving only water spray. Tween-20 (0.01%) was added as a surfactant to all spray solutions. Two foliar sprays were applied at fruit set and one month thereafter. Foliar sprays were applied using a foot-operated sprayer to ensure uniform coverage of the tree canopy, and 5 L of the respective nutrient solution or water (control) was applied per tree. All trees were maintained under uniform cultural practices throughout the experimental period (**Table 2**). To regulate cropping and avoid rainy-season fruiting, one-leaf-pair shoot pruning was carried out uniformly on all trees during the last week of April, following standard crop regulation practices (Tiwari and Lal, 2007; Mishra and Singh, 2022).

**Table 2:** Details of foliar potassium and calcium treatments applied in guava.

Symbol	Treatments	Concentrations (%)	Nutrient applied tree^-1^ (g)
T_0_	Control (spraying with plain water)	–	–
T_1_	Potassium nitrate (KNO_3_)	1.0	50
T_2_	Potassium nitrate (KNO_3_)	2.0	100
T_3_	Potassium nitrate (KNO_3_)	3.0	150
T4;	Potassium sulphate (K_5_4;)	1.0	50
T_5_	Potassium sulphate (K_5_4;)	2.0	100
T_6_	Potassium sulphate (K_5_4;)	3.0	150
T_7_	Calcium nitrate [Ca(NO_3_)_2_]	0.5	25
T_8_	Calcium nitrate [Ca(NO_3_)_2_]	1.0	50
T_9_	Calcium nitrate [Ca(NO_3_)_2_]	1.5	75
T_10_	Calcium sulphate (CaSO4;)	0.5	25
T_11_	Calcium sulphate (CaSO4;)	1.0	50
T_12_	Calcium sulphate (CaSO4;)	1.5	75

### Leaf growth parameters, chlorophyll content, and nutrient analysis

Ten fully expanded leaves per tree from the current-season shoots were tagged to record leaf length, breadth, and leaf area. These leaves were collected at the fruit maturity stage for subsequent data recording. Total chlorophyll content was estimated in fresh leaf samples using a double-beam spectrophotometer at wavelengths of 645 and 663 nm following the method of Lightenthaler (1987). For leaf nutrient analysis, 25–30 fully expanded leaves per replication borne at the fifth position from the base of the current-season shoot were collected from the mid-canopy zone, following the standardized leaf sampling protocol for guava described by Kumar and Pandey (1979). Sampling was conducted both before foliar application and after the termination of the experiment. The samples were washed thoroughly, oven-dried at 65 ± 5 °C for 72 h, and ground into a fine powder. Nitrogen content was determined using the Kjeldahl method, while phosphorus, potassium, calcium, and sulphur were estimated after di-acid digestion following standard analytical procedures (Jackson, 1973; Mishra et al., 2022).

### Fruit drop, fruit cracking, and yield assessment

Fruit drop percentage was calculated by recording the number of dropped fruits relative to the initial fruit set. Fruit cracking percentage was determined following the procedure described by Verner (1957). At harvest, total fruit number and fruit weight per tree were recorded. Fruit yield was expressed as kg tree^-^;¹ and converted to t ha^-^;¹ based on the plant population per hectare using the following formula:


$$\text{Yield }({\text{t ha}}^{-1})=\frac{{\text{Number of trees ha}}^{-1}\;\times \text{Yield }({\text{kg tree}}^{-1})}{1000}$$

Where, 1000 is the conversion factor used to convert kilograms to tonnes.

### Fruit physical and biochemical attributes

Fruit samples were collected at physiological maturity at harvest, and representative fruits from each experimental tree were used for physical and biochemical analyses. Fruit physical parameters, including fruit length, breadth, weight, volume, flesh thickness, and pulp and seed weight, were measured using digital calipers and an electronic balance. Fruit shape index was calculated as the ratio of fruit length to diameter. Biochemical attributes such as total soluble solids (TSS), titratable acidity, total sugars, reducing sugars, non-reducing sugars, pectin, and ascorbic acid were determined following standard AOAC methods (AOAC, 2023).

### Statistical and multivariate analyses

Data were analyzed using analysis of variance (ANOVA) under a randomized block design (RBD). When treatment effects were significant, mean comparisons were performed using Tukey’s honestly significant difference (HSD) test at p ≤ 0.05 following the procedure of Gomez and Gomez (1984). Percentage data (fruit cracking incidence) were arcsine square-root transformed prior to analysis to stabilize variance, although original means are presented for clarity. Multivariate analyses were performed to explore trait relationships and treatment similarities. Hierarchical clustering was conducted using standardized data with Euclidean distance and Ward’s linkage method. Partial least squares regression (PLSR) was employed to model the relationship between leaf physiological and nutrient traits and fruit quality attributes after z-score normalization. Partial Least Squares Regression (PLSR) was performed using standardized data to model the relationship between leaf traits (predictor matrix, X) and fruit quality parameters (response matrix, Y). The optimal number of latent components was selected using cross-validation. Model performance was assessed using R² and RMSE and Variable Importance in Projection (VIP) scores were calculated to evaluate predictor contribution. The PLSR model can be expressed as:


Y=XB+E

where X represents the standardized predictor matrix, B denotes the regression coefficient matrix derived from latent components, and E represents the residual error.

Statistical and graphical analyses were performed using Python-based packages (Seaborn and Matplotlib).

## Results

### Leaf growth, chlorophyll content, and nutrient status

Foliar application of potassium (K) and calcium (Ca) significantly influenced leaf growth parameters, chlorophyll content, and post-treatment leaf nutrient composition compared with the untreated control (**Table 3**). Prior to treatment application, leaf nutrient concentrations did not differ significantly among treatments. Among potassium treatments, KNO_3_ at 2.0% (T_2_) recorded the highest leaf length (12.68 cm), breadth (6.88 cm), and leaf area (72.25 cm²), representing increases of 32.4%, 37.1%, and 14.4%, respectively, over the control. This treatment also produced the highest chlorophyll content (3.60 mg g^-^;¹), followed closely by KNO_3_ at 3.0% (T_3_). Ca(NO_3_)_2_ at 1.5% (T_9_) resulted in comparable leaf growth responses, with leaf area (72.16 cm²) and chlorophyll content (3.23 mg g^-^;¹) statistically at par with T_2_. Post-treatment leaf nitrogen concentration (1.99%) increased significantly under Ca(NO_3_)_2_ at 1.5% (T_9_), while potassium concentration (1.05%) was highest under KNO_3_ at 3.0% (T_3_). Calcium content increased markedly under calcium-based treatments, with Ca(NO_3_)_2_ at 1.5% (T_9_) recording the highest value (0.76%). Sulphur concentration was enhanced primarily under sulphate-based treatments. Phosphorus content remained statistically non-significant both before and after treatment application.

**Table 3:** Effects of foliar potassium and calcium treatments on leaf morphological traits, chlorophyll content, and leaf nutrient concentrations of guava before and after application.

Treatments	Leaf length (cm)	Leaf breadth (cm)	Leaf area (cm²)	Chlorophyll (mg g^-1^)	N (%)		P (%)		K (%)		Ca (%)		S (%)	
					Before	After	Before	After	Before	After	Before	After	Before	After
T_0_- Control	9.58^f^	5.02 ^e^	63.18 ^g^	2.79 ^e^	1.98^a^	1.70 ^a^	0.20^a^	0.15^a^	0.93 ^a^	0.90 ^a^	0.70 ^a^	0.64 ^a^	0.20 ^a^	0.18 ^a^
T_1_- KNO_3_ 1.0%	9.97^ef^	5.53^bcde^	65.38^efg^	3.55^ab^	2.04^a^	1.90^a^	0.19^a^	0.13^a^	0.95^a^	1.00^a^	0.69^a^	0.63^a^	0.22^a^	0.19^a^
T_2_- KNO_3_ 2.0%	12.68^a^	6.88^a^	72.25^a^	3.60^a^	2.00^a^	1.92^a^	0.21^a^	0.15^a^	0.92^a^	1.02^a^	0.72^a^	0.64^a^	0.22^a^	0.20^a^
T_3_- KNO_3_ 3.0%	11.78^abcd^	6.20^abcde^	70.83^ab^	3.58^ab^	2.03^a^	1.97^a^	0.18^a^	0.13^a^	0.95^a^	1.05^a^	0.68^a^	0.63^a^	0.20^a^	0.18^a^
T_4_- K_5_4; 1.0%	9.58^f^	5.22^de^	63.92^fg^	3.33^abcde^	2.05^a^	1.80^a^	0.18^a^	0.12^a^	0.93^a^	0.96^a^	0.79^a^	0.62^a^	0.21^a^	0.22^a^
T_5_- K_5_4;2.0%	9.85^ef^	5.33^cde^	64.50^efg^	3.45^abcd^	1.97^a^	1.78^a^	0.22^a^	0.14^a^	0.92^a^	1.00^a^	0.71^a^	0.65^a^	0.22^a^	0.22^a^
T_6_- K_5_4; 3.0%	10.15^ef^	5.60^abcde^	66.60^def^	3.51^abc^	1.99^a^	1.80^a^	0.20^a^	0.14^a^	0.93^a^	1.04^a^	0.68^a^	0.63^a^	0.20^a^	0.22^a^
T_7_- Ca(NO_3_)_2_ 0.5%	10.78^cdef^	5.90^abcde^	68.89^bcd^	2.98^bcde^	1.96^a^	1.82^a^	0.19^a^	0.12^a^	0.94^a^	0.92^a^	0.70^a^	0.70^a^	0.19^a^	0.17^a^
T_8_- Ca(NO_3_)_2_ 1.0%	11.93^abc^	6.38^abcd^	70.97^ab^	3.19^abcde^	2.02^a^	1.95^a^	0.21^a^	0.15^a^	0.95^a^	0.95^a^	0.68^a^	0.73^a^	0.21^a^	0.18^a^
T_9_- Ca(NO_3_)_2_ 1.5%	12.65^ab^	6.75^ab^	72.16^a^	3.23^abcde^	2.04^a^	1.99^a^	0.19^a^	0.13^a^	0.94^a^	0.97^a^	0.70^a^	0.76^a^	0.20^a^	0.19^a^
T_10_- CaSO4; 0.5%	10.37^def^	5.87^abcde^	67.26^cde^	2.86^de^	2.00^a^	1.78^a^	0.20^a^	0.13^a^	0.92^a^	0.97^a^	0.71^a^	0.73^a^	0.20^a^	0.20^a^
T_11_- CaSO4; 1.0%	11.23^bcde^	6.12^abcde^	69.87^abc^	2.92^cde^	2.02^a^	1.78^a^	0.19^a^	0.14^a^	0.95^a^	0.99^a^	0.69^a^	0.73^a^	0.21^a^	0.22^a^
T_12_- CaSO4; 1.5%	11.95^abc^	6.55^abc^	71.85^ab^	3.02^abcde^	2.05^a^	1.85^a^	0.21^a^	0.15^a^	0.93^a^	1.00^a^	0.68^a^	0.75^a^	0.20^a^	0.22^a^
C.D. at 5%	0.679	0.553	2.940	0.126	NS	0.132	NS	NS	NS	0.108	NS	0.041	NS	0.012
SE(m) ±	0.231	0.188	1.001	0.043	0.024	0.045	0.004	0.005	0.015	0.037	0.12	0.013	0.003	0.003

The differences between the means indicated by different letters in the same column are significant at the *p* < 0.05 level.

### Fruit physical attributes and biochemical quality

Foliar nutrient treatments significantly improved fruit physical characteristics and biochemical quality attributes compared with the control (**Table 4**). Fruit length and breadth were maximized under Ca(NO_3_)_2_ at 1.5% (T_9_), whereas the highest flesh thickness (5.19 cm), fruit weight (488.52 g), and fruit volume (481.17 cm³) were recorded under KNO_3_ at 2.0% (T_2_), representing increases of 27.5%, 30.8%, and 20.5%, respectively, over the control. Fruit shape index and specific gravity did not differ significantly among treatments. Pulp weight and pulp-to-seed ratio were significantly enhanced by potassium treatments. KNO_3_ at 3.0% (T_3_) produced the highest pulp-to-seed ratio (107.55), followed by KNO_3_ at 2.0% (T_2_). Seed weight showed comparatively minor variation across treatments. Fruit biochemical quality parameters responded positively to foliar nutrient application. The highest total soluble solids (13.43°Brix), total sugars (8.16%), reducing sugars (5.67%), and ascorbic acid content (120.24 mg 100 g^-^;¹ pulp) were recorded under KNO_3_ at 2.0% (T_2_). Titratable acidity (0.38%) was lowest under KNO_3_ at 3.0% (T_3_). Pectin content was significantly influenced by calcium treatments, with Ca(NO_3_)_2_ at 1.5% (T_9_) producing the highest pectin concentration (1.18%). Non-reducing sugars did not differ significantly among treatments.

**Table 4:** Effects of foliar potassium and calcium treatments on fruit physical and biochemical attributes of guava.

Treatments	Fruit length (cm)	Fruit breadth (cm)	Shape index	Flesh thickness (cm)	Fruit weight (g)	Fruit volume (cm³)	Specific gravity (g cm^-3^)	Pulp weight (g)	Seed weight (g)	Pulp: Seed	TSS (°B)	Acidity (%)	Ascorbic acid (mg 100 g^-1^)	Total sugars (%)	Reducing sugars (%)	Non-reducing sugars (%)	Pectin (%)
T_0_- Control	7.89^e^	7.83^d^	1.01^a^	4.07^b^	373.52^j^	399.37^j^	0.94^a^	369.54^j^	3.98^e^	72.85^f^	10.13^e^	0.58^a^	98.13^h^	6.89^a^	4.52^c^	2.60^a^	0.91^a^
T_1_- KNO_3_ 1.0%	9.38^abc^	8.62^abcd^	1.09^a^	4.71^ab^	444.91^d^	447.47^d^	0.98^a^	440.17^d^	4.75^abcd^	88.58^cd^	12.93^a^	0.42^a^	117.86^abc^	7.94^a^	5.52^ab^	2.70^a^	1.08^a^
T_2_- KNO_3_ 2.0%	9.52^a^	9.43^a^	1.01^a^	5.19^a^	488.52^a^	481.17^a^	1.02^a^	483.95^a^	4.57^cde^	105.93^a^	13.43^a^	0.40^a^	120.24^a^	8.16^a^	5.67^a^	2.77^a^	1.05^a^
T_3_- KNO_3_ 3.0%	9.48^ab^	9.11^ab^	1.04^a^	5.02^ab^	488.48^a^	482.97^a^	1.01^a^	483.98^a^	4.50^cde^	107.55^a^	13.00^a^	0.38^a^	119.05^ab^	8.08^a^	5.64^a^	2.72^a^	1.03^a^
T_4_- K_2_SO_4_ 1.0%	8.25^de^	7.87^d^	1.05^a^	4.09^b^	382.09^1^	402.07^1j^	0.95^a^	377.17^1^	4.93^abcd^	76.56^ef^	11.40^bcde^	0.49^a^	103.74^g^	7.18^a^	4.77^c^	2.65^a^	0.94^a^
T_5_- K_2_SO_4_ 2.0%	8.57^cde^	8.22^bcd^	1.04^a^	4.38^ab^	398.82^fg^	414.87^fg^	0.96^a^	393.80^fg^	5.03^abc^	78.37^e^	11.17^cde^	0.54^a^	109.57^f^	7.25^a^	4.84^bc^	2.65^a^	0.97^a^
T_6_- K_2_SO_4_ 3.0%	8.61^bcde^	8.38^bcd^	1.03^a^	4.51^ab^	405.40^f^	420.63^ef^	0.97^a^	400.74^f^	4.66^bcde^	85.92^d^	11.57^bcd^	0.49^a^	110.71^ef^	7.54^a^	5.14^abc^	2.66^a^	1.01^a^
T_7_- Ca(NO_3_)_2_ 0.5%	8.40^de^	8.06^cd^	1.05^a^	4.25^ab^	395.15^gh^	411.57^gh^	0.96^a^	389.62^gh^	5.35^ab^	92.76^bc^	12.20^abc^	0.45^a^	113.48^de^	7.58^a^	5.14^abc^	2.69^a^	1.14^a^
T_8_- Ca(NO_3_)_2_ 1.0%	9.45^abc^	8.92^abc^	1.06^a^	4.96^ab^	459.40^c^	459.17^c^	1.00^a^	454.06^c^	5.38^a^	84.89^d^	12.20^abc^	0.47^a^	115.07^cd^	7.65^a^	5.24^abc^	2.67^a^	1.17^a^
T_9_- Ca(NO_3_)_2_ 1.5%	9.53^a^	9.46^a^	1.03^a^	4.97^ab^	475.02^b^	474.27^b^	1.00^a^	469.73^b^	5.42^a^	87.31^d^	12.60^ab^	0.49^a^	116.13^bcd^	8.06^a^	5.63^a^	2.72^a^	1.18^a^
T_10_- CaSO_4_ 0.5%	8.41^de^	7.92^d^	1.06^a^	4.13^b^	390.69^h^	407.87^h1^	0.96^a^	386.16^h^	4.53^cde^	85.31^d^	10.30^de^	0.51^a^	101.04^gh^	7.04^a^	4.59^c^	2.68^a^	1.11^a^
T_11_- CaSO_4_ 1.0%	8.88^abcd^	8.47^bcd^	1.05^a^	4.58^ab^	418.81^e^	423.27^e^	0.99^a^	414.55^e^	4.26^de^	96.28^b^	10.60^de^	0.55^a^	101.62^g^	7.07^a^	4.65^c^	2.66^a^	1.13^a^
T_12_- CaSO_4_ 1.5%	9.42^abc^	8.78^abcd^	1.07^a^	4.84^ab^	450.72^d^	452.27^d^	1.00^a^	445.70^d^	5.02^abc^	88.92^cd^	10.87^de^	0.56^a^	103.16^g^	7.14^a^	4.73^c^	2.65^a^	1.15^a^
C.D. at 5%	0.264	0.300	0.037	0.314	17.646	15.270	NS	17.649	0.164	6.625	0.550	0.094	3.034	0.248	0.172	NS	0.066
SE(m) ±	0.090	0.102	0.013	0.107	6.010	5.201	0.017	6.011	0.056	2.257	0.187	0.032	1.033	0.884	0.059	0.086	0.022

The differences between the means indicated by different letters in the same column are significant at the *p* < 0.05 level.

### Fruit drop, fruit retention, and fruit cracking

**Figure 3:**
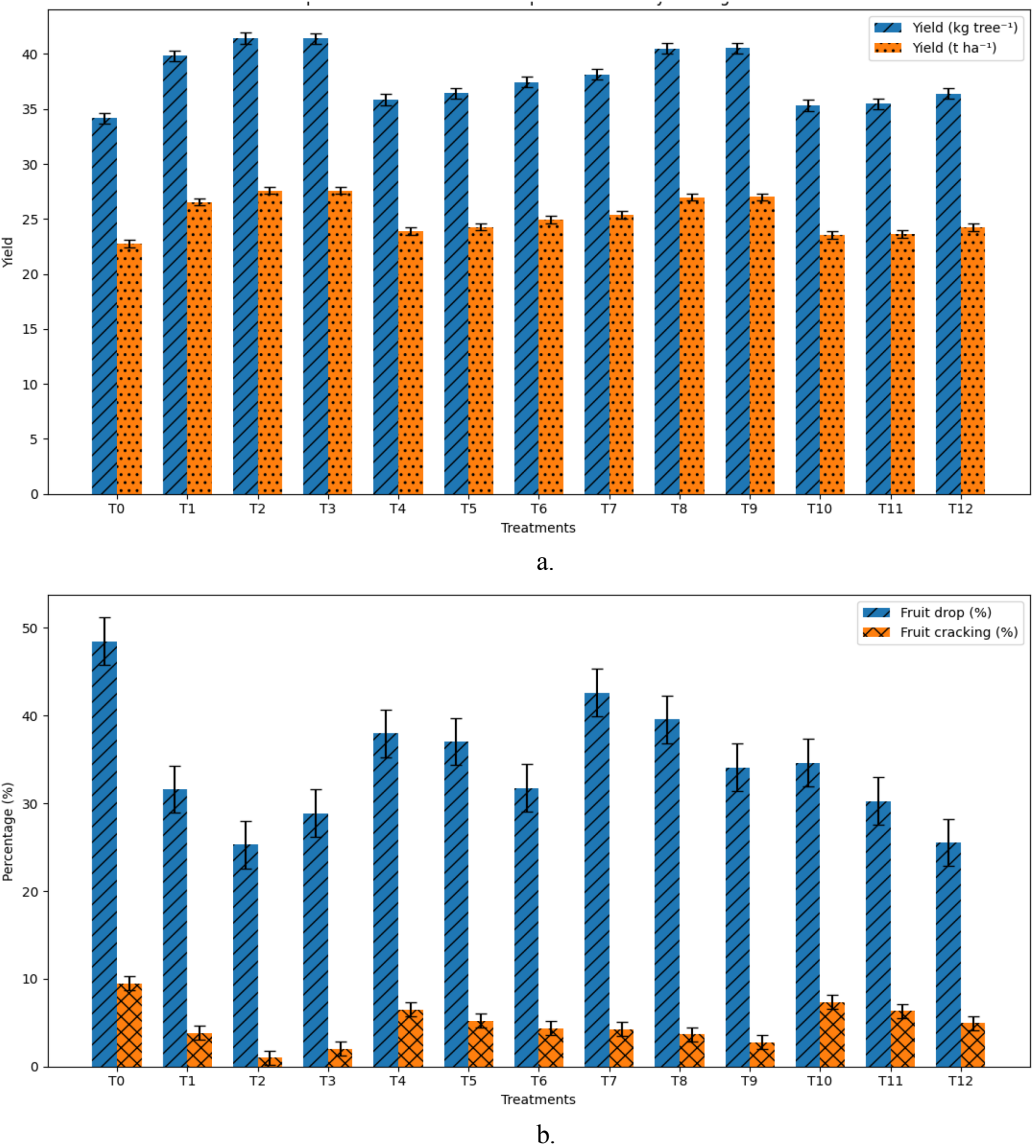
Effects of foliar potassium (K) and calcium (Ca) treatments (T0–T12) on guava yield and fruit physiological disorders (p<0.05): **(a)** fruit yield (kg tree^-^;¹) and fruit yield (t ha^-^;¹); **(b)** fruit cracking (%) and fruit drop (%).

Fruit drop and fruit cracking percentages varied significantly across treatments (**Figure 3**). The highest fruit drop (48.48%) and fruit cracking incidence (9.50%) were observed in the control. Foliar nutrient application markedly reduced both parameters. The lowest fruit drop (25.30%) and fruit cracking (1.00%) were recorded under KNO_3_ at 2.0% (T_2_), corresponding to reductions of 47.8% and 89.5%, respectively, compared with the control. CaSO_4_ at 1.5% (T_12_) and KNO_3_ at 3.0% (T_3_) also significantly reduced fruit drop and cracking, though to a lesser extent. Fruit retention showed an inverse trend, with the highest retention (74.70%) observed under KNO_3_ at 2.0% (T_2_).

### Fruit yield response

Fruit yield per tree and per hectare differed significantly among treatments (**Figure 3**). The lowest yield was recorded in the control (34.16 kg tree^-^;¹; 22.75 t ha^-^;¹). All potassium and calcium treatments increased yield relative to the control. Maximum yield was obtained with KNO_3_ at 2.0% (T_2_), which recorded 41.44 kg tree^-^;¹ and 27.60 t ha^-^;¹, representing a 21.36% increase over the control. This treatment was statistically at par with KNO_3_ at 3.0% (T_3_). Ca(NO_3_)_2_ at 1.5% (T_9_) also produced a significant yield increase (40.55 kg tree^-^;¹; 26.98 t ha^-^;¹), while sulphate-based treatments showed comparatively moderate yield enhancement.

### Hierarchical clustering of fruit loss parameters

**Figure 4:**
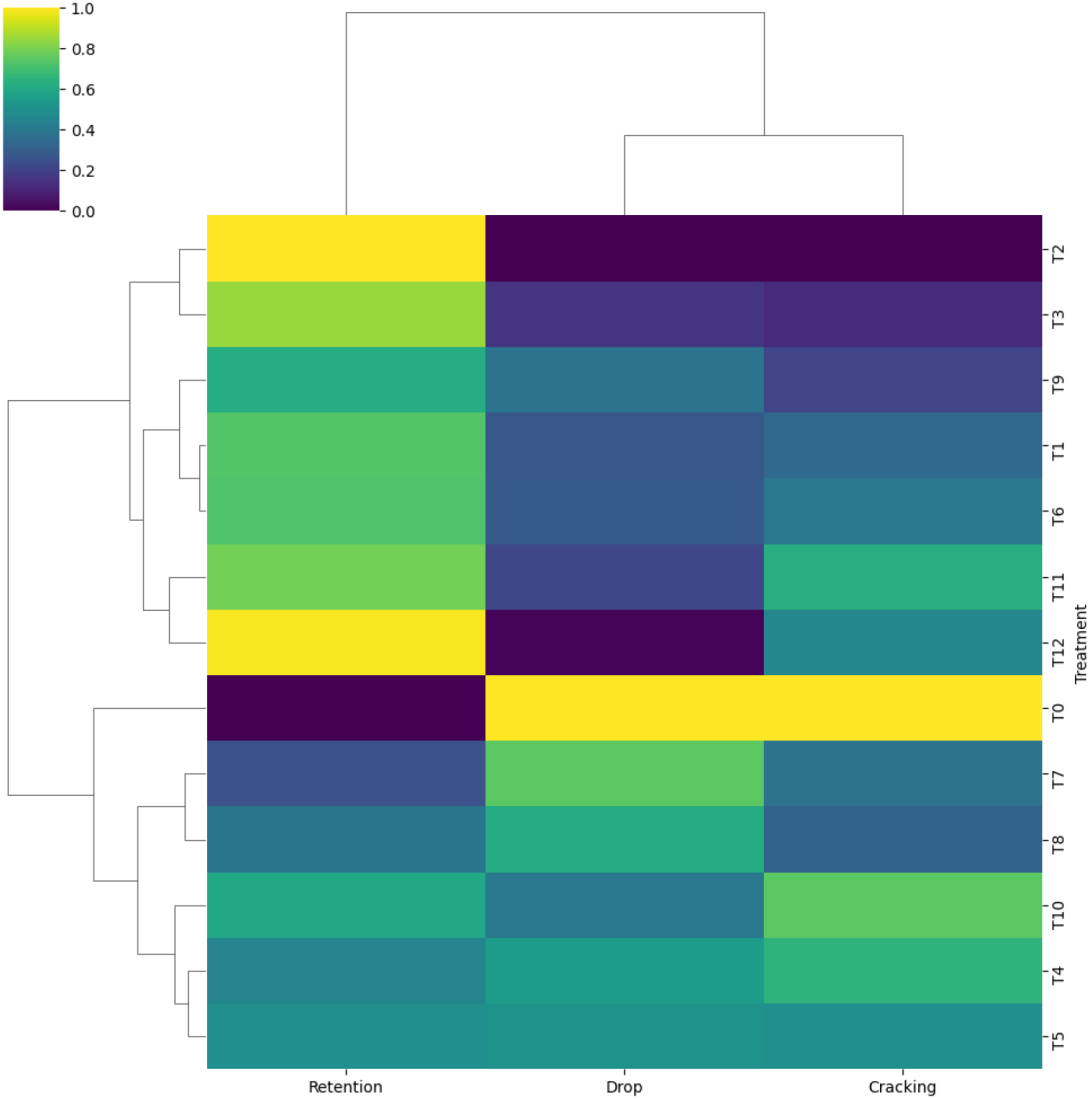
Heatmap with hierarchical clustering of fruit retention, fruit drop, and fruit cracking (%) under different foliar nutrient treatments, illustrating treatment-wise similarity based on normalized values.

Hierarchical cluster analysis based on fruit retention, fruit drop, and fruit cracking segregated the treatments into three distinct clusters (**Figure 4**). The first cluster comprised T_2_, T_3_, T_9_, T_1_, T_6_, T_11_, and T_12_, which were generally associated with higher fruit retention and comparatively lower fruit drop and cracking. T_2_ and T_12_ were grouped within the cluster associated with higher yield and fruit quality traits. The second cluster included treatments exhibiting intermediate responses, while the untreated control formed a separate cluster characterized by high fruit drop and cracking and low fruit retention. Trait-wise clustering further indicated an inverse association between fruit retention and fruit drop, whereas fruit cracking showed a partial association with fruit drop.

### Partial least squares regression analysis

**Figure 5:**
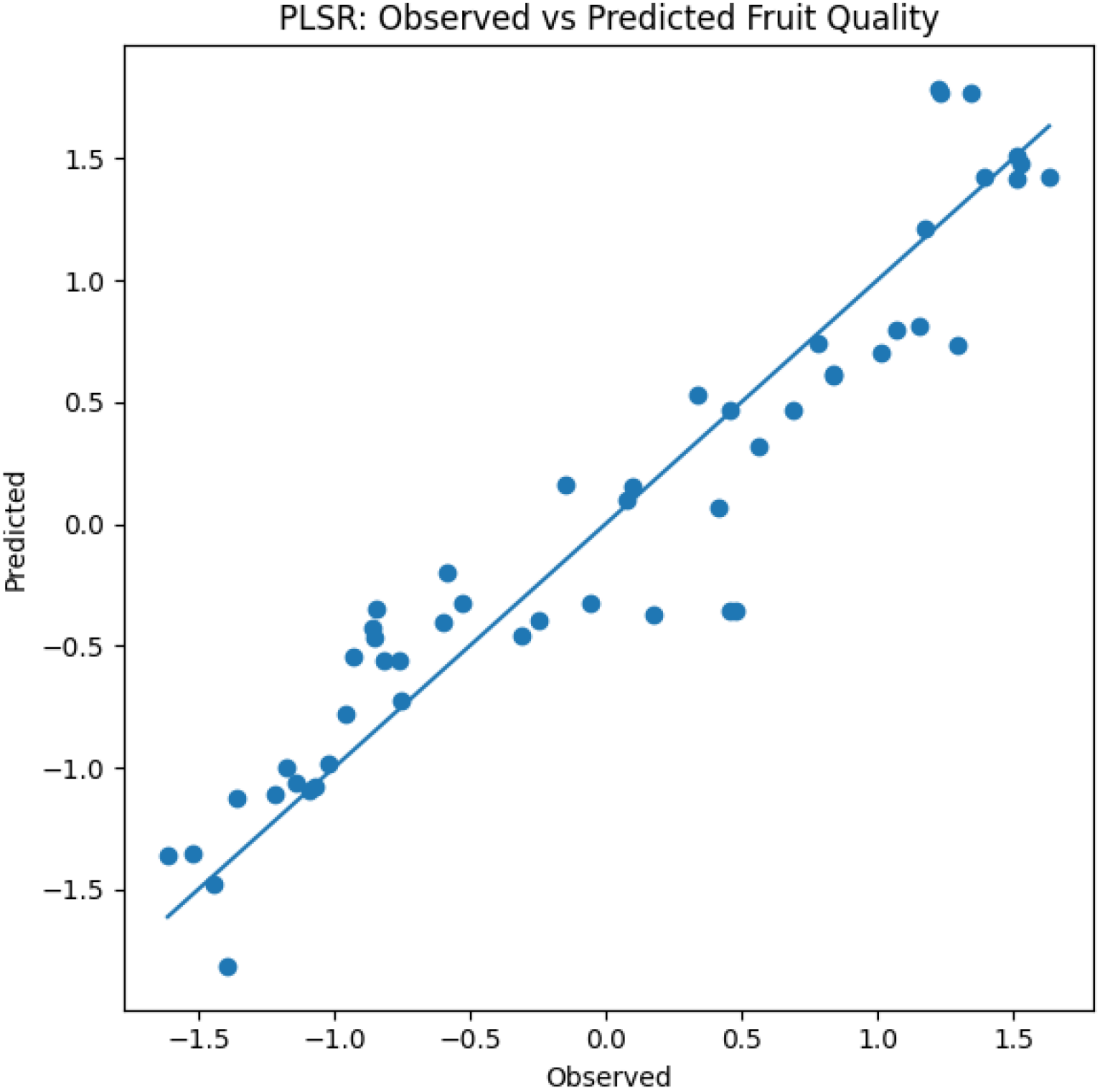
Observed vs predicted fruit quality values derived from PLSR showing strong model fit and prediction accuracy (R² = 0.877). The predictor matrix (X) consisted of leaf growth and nutrient variables measured after foliar application, including leaf length, leaf breadth, leaf area, chlorophyll content, and leaf nitrogen (N), potassium (K), calcium (Ca), and sulphur (S) concentrations. The response matrix (Y) comprised key fruit quality attributes, namely fruit weight, total soluble solids (TSS), ascorbic acid content, and total sugars.

Partial Least Squares Regression (PLSR) was conducted to evaluate the predictive relationship between leaf physiological traits and fruit quality parameters. The predictor matrix (X) consisted of leaf growth and nutrient variables measured after foliar application, including leaf length, leaf breadth, leaf area, chlorophyll content, and leaf nitrogen (N), potassium (K), calcium (Ca), and sulphur (S) concentrations. The response matrix (Y) comprised key fruit quality attributes, namely fruit weight, total soluble solids (TSS), ascorbic acid content, and total sugars. Prior to analysis, all variables were standardized using z-score normalization to eliminate scale differences. The optimal number of latent components was determined through cross-validation, and two components were retained based on maximum explained variance and model stability. Model performance was evaluated using the coefficient of determination (R²) and root mean square error (RMSE). The model exhibited strong predictive ability (R² = 0.877; RMSE = 0.31), indicating that approximately 87.7% of the variability in fruit quality parameters was explained by leaf physiological and nutrient characteristics. **Figure 5** presents the relationship between observed and predicted standardized fruit quality values derived from the PLSR model. The X-axis represents observed standardized fruit quality values, whereas the Y-axis represents model-predicted values. The close alignment of data points along the 1:1 regression line confirms good model fit and prediction accuracy. Variable Importance in Projection (VIP) scores were computed to identify influential predictors. Nitrogen (VIP = 1.38), chlorophyll (VIP = 1.21), leaf length (VIP = 1.06), and leaf breadth (VIP = 1.02) were identified as major contributors (VIP > 1), while potassium, calcium, and sulphur showed comparatively lower influence.

### Correlation among physiological, yield, and quality traits

Pearson correlation analysis among physical and biochemical traits revealed several significant associations (n = 13; p≤ 0.05). Strong positive correlations were observed between TSS and total sugars (r = 0.99), leaf area and fruit weight (r = 0.85), fruit weight and yield (r = 0.85), and total chlorophyll content and ascorbic acid (r = 0.83). Total chlorophyll content also showed positive associations with yield, TSS, and total sugars. The correlogram indicated that increased leaf area was associated with greater fruit weight and improved quality parameters. This suggested that enhanced leaf surface and chlorophyll content may contribute to greater assimilate production, thereby supporting fruit development and quality enhancement. Conversely, fruit cracking exhibited strong negative correlations with fruit weight (r = −0.90) and TSS (r = −0.94). Titratable acidity was negatively correlated with TSS (r = −0.92) and ascorbic acid (r = −0.90). Only correlations remaining significant after adjustment for multiple comparisons were interpreted. The observed correlation pattern indicated that vegetative traits such as leaf area and chlorophyll content are positively associated with economically important fruit attributes, whereas physiological disorders like fruit cracking are negatively associated with fruit quality and yield parameters.

## Discussion

### Foliar potassium and calcium modulate leaf physiology and nutrient status

The soil of the experimental site was moderately fertile with adequate potassium but relatively lower calcium availability. This nutrient status justified the evaluation of foliar potassium and calcium supplementation to enhance nutrient balance and improve fruit yield and quality in guava. Foliar application can be particularly beneficial under such conditions where soil calcium availability may limit optimal fruit development (Fernández and Eichert, 2009). The observed enhancement of leaf growth parameters and chlorophyll content following foliar potassium (K) and calcium (Ca) application confirms the central role of these nutrients in regulating vegetative physiology during the reproductive phase (**Table 3**). Potassium nitrate at 2.0% produced the most pronounced increase in leaf area and chlorophyll content, consistent with the function of K as a key activator of photosynthetic enzymes, regulator of stomatal conductance, and facilitator of assimilate transport (Tiwari, 2005; Nessem, 2019; Burstrom, 2008; El-Salhy et al., 2017). Comparable improvements under calcium nitrate treatments indicate that Ca-mediated stabilization of cell walls and membranes supports leaf expansion and chlorophyll maintenance (Taylor, 2003; Dolatabadian et al., 2013). The increase in post-treatment leaf nitrogen under calcium nitrate suggests improved nitrogen assimilation or retention, likely due to nitrate-derived N supply and Ca-mediated membrane integrity. The elemental composition of leaves remained relatively stable across treatments (**Table 3**), indicating that foliar agrochemical applications had only a limited influence on overall leaf mineral concentrations. Similar observations have been reported in other fruit crops such as apple, cherry, and currant, where foliar treatments resulted in only minor changes in leaf nutrient status. A slight decrease in leaf potassium concentration observed in the present study may be attributed to the translocation of potassium from leaves to developing fruits during the reproductive stage, reflecting the strong sink demand of fruits for this mobile nutrient. Comparable patterns of potassium redistribution from vegetative tissues to fruits have been reported in several fruit and berry crops (Elbert et al., 2002; Roeva, 2018; Roeva et al., 2018; Panfilova et al., 2024; Jha et al., 2025). The limited response of phosphorus across treatments aligns with previous reports showing restricted foliar P mobility and winter-season root uptake constraints (Fernández et al., 2013; Olsen, 1954). Together, these results indicate that foliar K and Ca application primarily alters leaf physiological competence and nutrient balance rather than uniformly increasing all macronutrients.

### Fruit physical growth and quality are differentially regulated by K and Ca

Improvements in fruit size, weight, and flesh thickness following foliar nutrient application demonstrate the downstream effect of enhanced leaf physiological status on sink development (**Table 4**). Potassium nitrate at 2.0% produced the highest fruit weight and volume, supporting the role of K in osmotic regulation, carbohydrate translocation, and cell enlargement during fruit growth (Solhjoo et al., 2017; Yeshitela et al., 2005). In contrast, calcium nitrate at 1.5% maximized fruit length and breadth, consistent with the involvement of Ca in cell division and structural reinforcement during early fruit development (Korkmaz and Askin, 2015; Muengkaew et al., 2018; Ali et al., 2019; Ranjbar et al., 2020; El-Motaium et al., 2025). The pronounced increase in pulp weight and pulp-to-seed ratio under potassium treatments reflects preferential allocation of assimilates toward edible tissue, a response previously reported in guava and other fruit crops following K supplementation (Burhan and AL-Taey, 2003; Kumar et al., 2015). Calcium-driven enhancement of pectin content corroborates its established role in pectin cross-linking and cell wall strengthening, contributing to improved textural quality (Dorria, 2001; Khalaj et al., 2017). These distinct yet complementary effects highlight nutrient-specific regulation of fruit structural and compositional traits.

### Regulation of fruit biochemical attributes by foliar nutrition

The increase in total soluble solids, sugars, and ascorbic acid under potassium nitrate treatments indicates enhanced carbohydrate synthesis and translocation from source leaves to developing fruits. Potassium is known to facilitate phloem loading and sugar transport, thereby increasing sugar accumulation and sweetness in fruits (Tiwari, 2005; Solhjoo et al., 2017; Muengkaew et al., 2018). The concurrent reduction in titratable acidity suggests a shift in carbon partitioning from organic acid pools toward soluble sugars, a response widely reported in potassium-fertilized fruit crops (Dhatt et al., 2005; Khalaj et al., 2017). Calcium-mediated increases in pectin content without substantial changes in soluble sugars indicate that Ca primarily influences structural and storage polysaccharides rather than sugar metabolism (Dorria, 2001; Dong et al., 2025). This differential regulation explains why potassium treatments predominantly enhanced sweetness and ascorbic acid, whereas calcium treatments contributed more strongly to firmness-related quality traits (Solhjoo et al., 2017; Lester et al., 2005; White and Broadley, 2003). Such nutrient-specific biochemical responses reinforce the importance of balanced foliar nutrition during fruit development.

### Fruit drop and cracking are strongly mitigated by coordinated K and Ca supply

The marked reduction in fruit drop and fruit cracking under potassium nitrate at 2.0% demonstrates the effectiveness of foliar nutrition in mitigating major physiological losses in guava (**Figure 3**). Reduced fruit drop may be attributed to improved nutrient availability during early fruit development, supporting hormonal balance and sink strength, thereby limiting abscission (Wood et al., 2010; Hardiyanto and Friyanti, 2019; Okba et al., 2021; Bons and Sharma, 2023). The strong suppression of fruit cracking aligns with the combined effects of potassium-mediated osmotic regulation and calcium-mediated reinforcement of peel cell walls (Huang et al., 2005; Dong et al., 2025). Calcium treatments also reduced cracking, albeit to a lesser extent than potassium nitrate, consistent with their primary role in cell wall stabilization rather than water balance regulation. Nevertheless, rapid cell expansion driven by K-mediated osmotic regulation, when not accompanied by adequate Ca-mediated structural reinforcement, may increase cracking risk under high humidity (Taylor, 2003). Previous studies have linked Ca accumulation in the fruit peel with reduced activity of cell wall–degrading enzymes, thereby enhancing resistance to cracking under fluctuating water conditions (Fan et al., 2023; Zhu et al., 2023; Wang et al., 2024). The present findings confirm that effective cracking control requires both structural reinforcement and balanced water relations. Thus, combined K–Ca treatment appeared to balance these processes, enhancing growth while mitigating cracking incidence.

### Yield enhancement reflects integrated physiological and nutritional effects

The significant yield increase observed under potassium nitrate at 2.0% reflects the cumulative effect of improved leaf physiology, enhanced fruit retention, reduced cracking, and superior fruit size. Similar yield responses to foliar K and Ca application have been reported in guava (Kumar et al., 2017), apple (Solhjoo et al., 2017), mango (Bibi et al., 2019), and citrus (Alva et al., 2006) particularly under conditions where root uptake is constrained. The comparatively moderate response of sulphate-based treatments suggests that nitrate forms may provide additional benefits through readily available nitrogen supply during critical growth stages.

### Multivariate analyses reveal trait-based regulation of fruit quality

**Figure 6:**
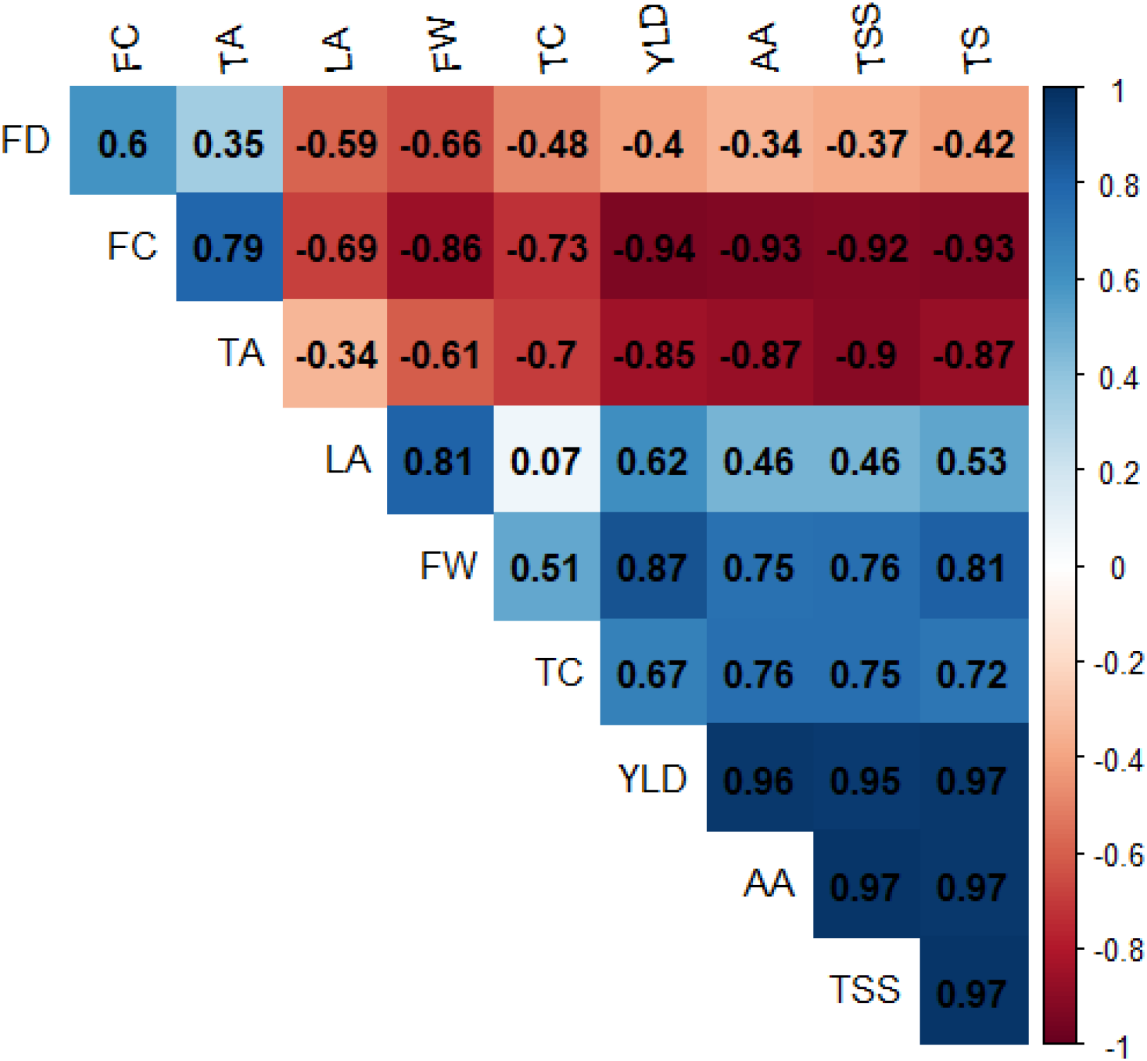
Pearson correlation matrix based on treatment mean values (n = 13) showing relationships among fruit physical and biochemical traits (P ≤ 0.05). Correlation coefficients (r) are displayed within each cell. Statistical significance was assessed at P ≤ 0.05 and adjusted using the Benjamini–Hochberg false discovery rate (FDR) correction. Red indicates positive correlations and blue indicates negative correlations. (FD, fruit drop; FC, fruit cracking; TA, titratable acidity; LA, leaf area; FW, fruit weight; TC, total chlorophyll; YLD, fruit yield; AA, ascorbic acid; TSS, total soluble solids).

Hierarchical clustering clearly separated treatments based on fruit retention, drop, and cracking, with potassium nitrate and selected calcium treatments grouping together due to their favorable trait combinations (**Figure 4**). The isolation of the control treatment emphasizes the magnitude of foliar nutrient effects on fruit stability. Partial least squares regression further demonstrated that leaf nitrogen content, chlorophyll concentration, and leaf size were the strongest predictors of fruit quality, highlighting the central role of source strength in determining sink performance (**Figure 5**). The lower VIP scores for K and Ca suggest that their effects on fruit quality are largely mediated through improvements in leaf physiological traits rather than direct accumulation alone. These findings support earlier reports linking enhanced photosynthetic capacity and nutrient status to improved fruit biochemical attributes (Fernández et al., 2013; Weng et al., 2022; Guo et al., 2023). Correlation analysis reinforced these relationships by revealing strong positive associations between leaf area, chlorophyll content, fruit weight, and yield, while fruit cracking showed strong negative correlations with fruit weight and TSS (Fan et al., 2023; Mishra et al., 2025a) (**Figure 6**). Together, these analyses provide a trait-based framework linking foliar nutrition, leaf physiology, and fruit quality outcomes.

## Conclusion

The results indicate that although the experimental soil was moderately fertile with adequate potassium, relatively lower calcium availability may limit optimal fruit development in guava. Under such conditions, foliar supplementation of potassium and calcium proved beneficial for improving nutrient balance, fruit yield, and fruit quality. The study demonstrates that nutrient imbalance during the reproductive stage can constrain guava productivity, and that foliar application of potassium and calcium helps alleviate these limitations through coordinated effects on leaf physiological status, nutrient dynamics, and fruit structural integrity. Among the treatments evaluated, potassium nitrate at 2.0% produced the highest yield along with improved fruit size, biochemical quality, and reduced fruit drop and cracking. These responses were associated with enhanced leaf nitrogen status, chlorophyll content, and leaf growth, highlighting the importance of source strength in supporting fruit sink development. Calcium-based treatments, particularly calcium nitrate at 1.5%, contributed to improved fruit structural traits such as pectin content and peel integrity, thereby reducing fruit cracking. Multivariate analyses further revealed strong relationships between leaf physiological traits and fruit quality attributes, supporting a trait-based understanding of nutrient-mediated regulation of fruit development. Overall, these findings provide a physiological basis for optimizing foliar potassium and calcium nutrition as a practical strategy to enhance yield and reduce fruit losses in guava under subtropical conditions.

## Data Availability

The datasets presented in this study can be found in online repositories. The names of the repository/repositories and accession number(s) can be found in the article/supplementary material.
